# How do responses vary between mothers and their daughters on measuring daughter’s self-rated health (SRH): a study among school-going adolescent girls in the primary setting of Varanasi, India

**DOI:** 10.1186/s13104-022-06174-1

**Published:** 2022-09-05

**Authors:** Ratna Patel, Dhananjay W. Bansod

**Affiliations:** Department of Public Health and Mortality Studies, International Institute for Population Sciences, Mumbai, India

**Keywords:** Self-rated health, Opinion difference, Adolescents, India

## Abstract

**Objective:**

How self-rated health (SRH) varies when the response on SRH is recorded from the respondent herself (adolescent girl) and her mother on her behalf. This study examines the prevalence of SRH among adolescent girls from her point of view as well as from her mother’s point of view. This insight could help us interpret the differences in opinion of girls and their mothers while measuring the girls’ self-rated health.

**Results:**

Almost one-fifth (19.4%) of the girls reported poor SRH. In contrast, only one in eight mothers (12.3%) could report their daughters under the category of poor SRH. Nearly one-third (76.5%) of the mothers reported their daughter’s SRH as good when daughters themselves rated poorly on SRH and another one-tenth (9.6%) reported their daughter’s SRH as poor when daughters themselves categorized in the good SRH category [χ^2^ = 9.900; p < 0.002]. More than 90 percent of the Rich and Middle wealth index women, women in the household with only daughters and no son, women whose husbands had higher education, women with higher secondary education, and non-working women visualized their daughter’s SRH as good when daughters themselves reported poor SRH.

**Supplementary Information:**

The online version contains supplementary material available at 10.1186/s13104-022-06174-1.

## Introduction

There is a widespread consensus in the available literature that self-rated health (SRH), measured as a one-item general health question, is one of the important health indicators with widespread applicability [1, 2]. Despite its widespread applicability, hardly any other measure of health is more poorly understood than self-rated health [1]. This measure is based on asking individuals to evaluate their health status and is being frequently employed in sociological health research since the 1950s [2–4]. The question on self-rated health is so prominent that the data on SRH are collected in major national and international surveys including World Value Survey, European value Survey, and Longitudinal Ageing Study in India, and recommended as a standard part of health surveys [5].

Self-rated health is a well-known and reliable indicator to measure the health status of the population [6], even in India [7, 8]. It has been studied widely across countries [9–17] and sub-populations including older adults [18–24], adults [25–28], and adolescents [29–33]. Somehow, studies examining factors associated with self-rated health among adolescents remained unearthed topic in the Indian context. Furthermore, how self-rated health varies when the response was recorded from the respondent herself (adolescent girl) and was asked from her mother on her behalf. This study is unique in the sense that the self-rated health was asked from adolescent girls and their mothers depicting the possible discordant between a mother and her daughter. Therefore, this study examines the prevalence of self-rated health among adolescent girls from her point of view as well as from her mother’s point of view. This insight could help us interpret the differences in opinion of girls and their mothers while measuring the girls’ self-rated health and also presents possible correlates of poor self-rated health among adolescent girls.

## Main text

### Data and methods

This study is based on the primary data collected in the Varanasi district of Uttar Pradesh, India, from October 2019 to February 2020. Nearly 350 adolescent girls and their mothers were personally interviewed. The purpose of the primary survey was to examine the self-rated health status of the adolescent girls and then compare the response with that of mothers’. While framing the question on self-rated health, it was hypothesized that mothers might not be well informed about their daughter’s self-rated health and therefore any deviation between the responses of mothers and daughters on self-rated health would provide discordance in mothers’ responses on self-rated health.

#### Sample size estimation

The adolescent consists of children in the age group 10–19 years of age. Adolescent can be divided into three various groups based on their age group namely; early adolescent (10–12 years), middle adolescent (13–16 years), and late adolescent (17–19 years). This study is based on middle and late-adolescent girls. The study was conducted on school-going girls (8th standard to 12th standard) in the age group between 13 and 19 years of age.

For taking prevalence, the number of literate girls in the urban area of Varanasi, as per census 2011, in the age group 13–19 are taken as the numerator and total girls in the age group 13–19 are taken as the denominator.

$${\text{p}} = \frac{{Number{\mkern 1mu} \,of\,{\mkern 1mu} literate\,{\mkern 1mu} girls\,{\mkern 1mu} in{\mkern 1mu} \,the\,{\mkern 1mu} age\,group\,{\mkern 1mu} 13{{{-}}}19\,years\,{\mkern 1mu} in\,{\mkern 1mu} urban{\mkern 1mu} \,Varanasi}}{{Total\,girls\,{\mkern 1mu} in\,{\mkern 1mu} the\,{\mkern 1mu} age\,group\,13{{{-}}}19\,years\,{\mkern 1mu} in\,{\mkern 1mu} urban{\mkern 1mu} \,Varanasi}}*100,$$

$${\text{p}}=\frac{103373}{120986}*100,$$

p = 85.44.

The sample size estimation for the study is done by using the formula developed by Cochran (1977). The formula is as follows:

$${\text{n}}=\frac{\left(z\right)2*p*q}{\left(d\right)2}$$

where, n = Required Sample Size; Z = 1.96 (95% level of confidence); p = 0.8544; q = 0.1456.

α = 0.05 (5% margin of error); **n = 191**.

By taking a non-response rate of 10 percent and a design effect of 1.5, the sample size was to be:

$${\text{n}} = {211}\,\,*\,\,{1}.{1}\,\,*\,\,{1}.{5} = {\mathbf{315}} \, {\mathbf{Individuals}}$$

So, nearly **350 adolescent girls** from the school were interviewed.

#### Sampling design

Varanasi district is subdivided into five zones for ease of administration. A total of ten schools were selected, two from each zone (Wards). Out of ten schools, five public and five private schools were selected. Two schools, one public, and one private school were selected from each zone (wards). From each school, a total of 35 students were interviewed. These 35 students were selected from classes 8–12th. From each class, 7 students were selected for the interview.

#### Selection of school

Varanasi city is divided into five zones and zones are further divided into wards. One ward was selected from each zone randomly. After selecting five wards, one from each zone, a complete public, and private school listing was carried out. Two schools, one private and one public school were selected from each ward randomly. If in case, a ward is not having either of public or private school, the next ward was selected randomly. If in case, a school is not interested in participating in the study, the next school was selected randomly.

#### Selection of respondents from school

From each class, seven students were selected by employing systematic random sampling. For sampling, a complete list of students was taken from the class attendance register. The mothers of the selected student were personally interviewed in their households.

#### Inclusion criteria

1. Girls aged 13–19 years of age; and
2. Girls studying in class from 8 to 12th.

#### Exclusion criteria

1. Disabled girls were not interviewed; and
2. Those girls whose mothers are not alive were not interviewed.

#### Outcome variable

Self-rated health was the primary outcome variable of this study. SRH was a dichotomous variable where 0 means ‘Good SRH’ and 1 means ‘Poor SRH.’ The exact wording of the question asked from the adolescent girls was “In general, how would you say your health is?” Similarly, the exact wording of the question asked from the mothers of adolescent girls was “In general, how would your daughter rate her health?” In both scenario, the SRH of the girl was asked, however from a different perspective. In the first case, a girl herself is reporting her SRH and in the second case, her mother is visualizing the SRH on her behalf. By doing so, we aimed at quantifying the mismatch in the response of SRH between mothers and their daughters.

#### Exposure variable

Exposure variables were divided into three groups; (1) Household Characteristics; Caste [Scheduled Castes/Scheduled Tribes (SC/ST), Others Backward Castes (OBC), and Others], Religion (Hindu and Non-Hindu), Wealth Index (Poorest, Poor, Middle, Rich, and Richest), and Composition of Children (Only daughter/no son, equal son and daughter, more son/less daughter, and more daughter/less son); (2) Parental characteristics; Father’s education level (No education, Primary, Secondary, Higher Secondary, and Higher Study), Mother’s education level (No education, Primary, Secondary, Higher Secondary, and Higher Study), Working status of father (Working and Not working), and Working status of mother (Working and Not working); and (3) Adolescent girl’s characteristics; Girl’s education level (8–10th and 11–12th) and Age of the girl (13–15 years, and 16–19 years).

#### Statistical analysis

The study uses bivariate analysis; and to depict the significance, a chi-square test was performed.

#### Ethical issues

The study proposal and survey questionnaires were approved by the Student Research Ethics Committee (SREC) of the institute. Written consent was taken from the individual respondents. Participation in the study was made voluntary, and participants were allowed to withdraw at any point during the interview if desired. Additional files 1 and 2 presents the adolescents’ and mothers’ questionnaires respectively.

### Results

Table 1 depicts the percentage distribution of the sample by selected background variables along with depicting the prevalence of poor SRH as reported by girls and as visualized by their mothers by background characteristics. Almost one-fifth (19.4%) of the girls reported poor SRH for them. In contrast, only one in eight mothers (12.3%) could visualize their daughters under the category of poor SRH. Reporting of poor SRH varies between girls and their mothers by almost all the background characteristics as depicted in Table 1. The stark differences in reporting SRH by girls and their mothers were noticed for higher wealth index and higher educational categories.

**Table 1:** Percentage distribution of the selected sample of the girls, bivariate distribution of SRH as reported by adolescent girls and their mothers, and difference in reporting in SRH by girls and their mothers

Column A	Column B	Column C	Column D	Column E	Column F
Background Characteristics	Total sample N (%)	Poor SRH as reported by girls (%)	Poor SRH as reported by girl’s mothers (%)	Absolute Difference in SRH (Girl’s SRH- mother’s SRH)	Percent change (Girl’s SRH- mother’s SRH/mother’s SRH) *100
				(Column C – ColumnD)	(Column E/Column D)*100
Household characteristics					
Caste					
SC/ST	69 (19.7)	24 (34.8)	21 (30.4)	4.4	14.5
OBC	170 (48.6)	26 (15.3)	17 (10)	5.3	53.0
Others	111 (31.7)	18 (16.2)	5 (4.5)	11.7	260.0
Religion					
Hindu	271 (77.4)	52 (19.2)	28 (10.3)	8.9	86.4
Non-Hindu	79 (22.6)	16 (20.3)	15 (19.0)	1.3	6.8
Wealth index					
Poorest	67 (19.1)	24 (35.8)	26 (38.8)	− 3.0	− 7.8
Poor	73 (20.9)	12 (16.4)	9 (12.3)	4.1	33.33
Middle	70 (20.0)	8 (11.4)	1 (1.4)	10	714.3
Rich	69 (19.7)	11 (15.9)	3 (4.4)	11.5	261.4
Richest	71 (20.3)	13 (18.3)	4 (5.6)	12.7	226.8
Composition of children					
Only daughter/ no son	39 (11.1)	2 (5.1)	5 (12.8)	− 7.7	− 60.2
Equal son and daughter	126 (36.0)	31 (24.6)	16 (12.7)	11.9	93.7
More son/less daughter	117 (33.4)	18 (15.4)	11 (9.4)	6	63.8
More daughter/less son	68 (19.4)	17 (25.0)	11 (16.2)	8.8	54.3
Parental characteristics					
Father education					
No education	53 (15.4)	12 (22.6)	13 (24.5)	− 1.9	− 7.8
Primary	54 (15.7)	16 (29.6)	11 (20.4)	9.2	45.1
Secondary	67 (19.4)	11 (16.4)	7 (10.5)	5.9	56.2
Higher secondary	65 (18.8)	10 (15.4)	9 (13.9)	1.5	10.8
Graduation and above	106 (30.7)	18 (17.0)	3 (2.8)	14.2	507.1
Mother education					
No education	97 (27.7)	27 (27.8)	16 (16.5)	11.3	68.5
Primary	59 (16.9)	6 (10.2)	6 (10.2)	0	0
Secondary	78 (22.3)	18 (23.1)	14 (18.0)	5.1	28.3
Higher secondary	83 (23.7)	13 (15.7)	5 (6.0)	9.7	161.7
Graduation and above	33 (9.4)	4 (12.1)	2 (6.1)	6	98.4
Working status of Father^a^					
Working	334 (96.8)	63 (18.9)	43 (12.9)	6	46.5
Not working	11 (3.2)	4 (36.4)	0 (0.0)	36.4	^b^
Working status of Mother					
Working	39 (11.1)	8 (20.5)	4 (10.3)	10.2	99.0
Not working	311 (88.9)	60 (19.3)	39 (12.5)	6.8	54.4
Adolescent girl’s characteristics					
Girl’s Educ.					
8–10th	210 (60.0)	41 (19.5)	25 (11.9)	7.6	63.9
11–12th	140 (40.0)	27 (19.3)	18 (12.9)	6.4	49.6
Age of the girl					
13–15 years	181 (51.7)	36 (19.9)	20 (11.1)	8.4	75.7
16–19 years	169 (48.3)	32 (18.9)	23 (13.6)	5.3	41.2
Total	350 (100)	68 (19.4)	43 (12.3)	7.1	57.7

*SRH* self-rated health, *SC* scheduled caste, *ST* scheduled tribe, *OBC*: other backward class

^a^Total is not 350 as fathers were not alive in these households

^b^Value is not available as category of non-working father has no sample for column D

Table 2 depicts the discordance in SRH as reported by girls and their mothers. Nearly one-third (76.5%) of the mothers visualized their daughter’s SRH as good when daughters themselves rated poorly on SRH and another one-tenth (9.6%) visualized their daughter’s SRH as poor when daughters themselves categorized in the good SRH category.

**Table 2:** Discordance in SRH as reported by daughters and their mothers

SRH from daughter’s perspective	SRH from mother’s perspective			Chi-square value
	Good SRH	Poor SRH	Total	
Good SRH	255 (90.4%)	27 (9.6%)	282 (100%)	χ^2^ = 9.900; p < 0.002
Poor SRH	52 (76.5%)	16 (23.5%)	68 (100%)	
Total	307 (87.7%)	43 (12.3%)	350 (100%)	

*SRH* self-rated health

Table 3 shows the response of mothers on visualizing the SRH of their daughters when their daughters rated their SRH as poor. A significant proportion of mothers from all the given background characteristics visualized their daughter’s SRH as good when daughters themselves rated poorly on SRH. More than 90% of the Rich and Middle wealth index women, women in the household with only daughters and no son, women whose husbands had higher education, women with higher secondary education, and non-working women visualized their daughter’s SRH as good when daughters themselves reported poor SRH.

**Table 3:** Response of mothers on SRH when daughters reported poor SRH for themselves by various background characteristics

Background Characteristics	Good SRH	Poor SRH	Chi-square value
Household characteristics			
Caste			
SC/ST	58.3	41.7	χ^2^ = 2.198; p < 0.139
OBC	84.6	15.4	χ^2^ = 0.9888; p < 0.320
Others	88.9	11.1	χ^2^ = 2.1799; p < 0.140
Religion			
Hindu	75.0	25.0	χ^2^ = 14.94; p < 0.00
Non-Hindu	81.2	18.8	χ^2^ = 0.007; p < 0.978
Wealth index			
Poorest	58.3	41.7	χ^2^ = 0.1289; p < 0.720
Poor	75.0	25.0	χ^2^ = 2.133; p < 0.144
Middle	100.0	0.0	χ^2^ = 0.1309; p < 0.717
Rich	90.9	9.1	χ^2^ = 0.708; p < 0.400
Richest	84.6	15.4	χ^2^ = 2.846; p < 0.092
Composition of children			
Only daughter/no son	100.0	0.0	χ^2^ = 0.310; p < 0.578
Equal son and daughter	71.0	29.0	χ^2^ = 9.895; p < 0.002
More son/less daughter	83.3	16.7	χ^2^ = 1.318; p < 0.251
More daughter/less son	76.5	23.5	χ^2^ = 0.904; p < 0.342
Parental Characteristics			
Father education			
No education	66.7	33.3	χ^2^ = 0.649; p < 0.420
Primary	56.3	43.7	χ^2^ = 7.662; p < 0.006
Secondary	72.7	27.3	χ^2^ = 3.982; p < 0.046
Higher Secondary	90.0	10.0	χ^2^ = 0.147; p < 0.702
Graduation and above	94.4	5.6	χ^2^ = 0.587; p < 0.444
Mother education			
No education	74.1	25.9	χ^2^ = 2.416; p < 0.120
Primary	83.3	16.7	χ^2^ = 0.309; p < 0.579
Secondary	66.7	33.3	χ^2^ = 3.761; p < 0.052
Higher Secondary	92.3	7.7	χ^2^ = 0.076; p < 0.783
Graduation and above	75.0	25.0	χ^2^ = 2.868; p < 0.090
Working status of Father^a^			
Working	74.6	25.4	χ^2^ = 0.550; p < 0.815
Not working	100.0	^a*^	^a**^
Working status of Mother			
Working	87.5	12.5	χ2 = 0.550; p < 0.815
Not working	75.0	25.0	χ2 = 10.523; p < 0.001
Adolescent girl’s characteristics			
Girl’s Educ.			
8–10th	78.1	21.9	χ^2^ = 4.903; p < 0.027
11–12th	74.1	25.9	χ^2^ = 5.099; p < 0.024
Age of the girl			
13–15 years	77.8	22.2	χ^2^ = 5.707; p < 0.017
16–19 years	75.0	25.0	χ^2^ = 4.356; p < 0.037
Total	52 (76.5)	16 (23.5)	χ^2^ = 9.900; p < 0.002

Numbers in each column depicts percentage of Good SRH and Bad SRH

*SRH* self-rated health, *SC* scheduled caste, *ST* scheduled tribe, *OBC* other backward class

^a^It depicts that the total number of fathers is not 350 as in some households, fathers were not alive

^a^*Due to non of the sample falling in the respective categories, values is not available

^a^**Chi-square value is not available as the corresponding category of variable does not have any sample for non-working class of fathers

### Discussion

This study examined the SRH among school-going adolescent girls by adopting a unique approach: probing the self-rated health of the girls by girls and their mothers. Firstly, a girl was asked to rate her health as either good or poor and then her mother was asked about how her daughter would rate her health. By doing so, this study explored the possible perception of mothers on how well they understand their daughter’s health. The study noticed that a significant proportion of mothers failed to understand their daughter’s perception of self-rated health. It is significantly evident from the findings that a large proportion of mothers visualized their daughter’s SRH as good when daughters themselves were categorized poorly on SRH. What could be the possible mechanisms and why mothers fail to recognize the health condition of their daughters is worth probing.

Family bonds have been characterized as an asset in health promotion intervention [34] and studies in the Indian context have clearly outlined the gender biases in the family towards the female gender from a health perspective [35]. This biasedness towards the female gender could be a possible attribute why mothers fail to correctly percept the poor SRH of their daughters as outlined in this study. In line with this discussion, the study also noted that in the case of an equal number of sons and daughters in the household, a significant proportion of mothers tend to visualize their daughter’s SRH as good when the daughter reported poor SRH. This finding weighs on a longstanding debate of gender inequality in households disfavouring females. There could also be another possible reason why mothers think that their daughters would rate their health as good when daughters rated their health as poor. Lundberg et al. [36] opined that boys as compared to girls are more likely to reap financial and emotional benefits from their parents, and this could be one of the plausible factors why in this study mothers failed to visualize poor SRH of their daughters.

A higher proportion of mothers from rich wealth quintile and higher education status visualized their daughters' SRH as good when daughters reported poor SRH. Highly educated mothers belonging to the rich wealth quintile are more likely to work and their career might demand long work hours leading to negligence in child care [37] and thereby it can be inferred that they might not be well aware of their daughter’s SRH as depicted in this study. Luthar and Latendresse [37] called this an antecedent of ‘*isolation from adults’* where among upper-middle-class families, adolescent children often left home alone for several hours each week, giving a sense of self-sufficiency to parents that could have several emotional repercussions to children. Social support from family, specifical parents, plays an imperative role in the life of the adolescents and also is a critical factor for them to rate their self-rated health [38], and it is understood that in affluent families children might feel neglected [39]. This feeling of neglect might have driven adolescents to rate their health as poor, while mothers might be under the impression that belonging to the rich wealth quintile automatically helps them to visualize their daughter’s health as good.

## Conclusion

A mother’s knowledge about children’s health is impromptu to the quality of care she provides to her children and therefore it becomes critical to seek improvements in mother’s involvement in the healthcare needs of their children. This study clearly outlined that mothers failed to visualise correct SRH for their daughters as discrepancies were noticed between mothers visualizing their daughter’s SRH and reporting of SRH by daughters themselves. The involvement of mothers in visualizing children’s health shall be promoted to avoid any serious complications. Mother-daughter relationships may be a potential asset to promote good self-rated health among adolescent girls. It is highly desirable to educate mothers on the importance of self-rated health of their daughters and therefore the importance of the mother-daughter relationship as a locus for health promotion is critical.

## Strengths of the study

We could not find a single study that examined the discordance in the perception of self-rated health between daughters and their mothers in the Indian context. That way, this is quite an untouched domain and may pave the way for future research. The response of daughters and their mothers on SRH were collected following the guidelines of the widely used KIDSCREEN-52 scale.

## Limitations of the study

Despite above-mentioned strengths, the study has some potential limitations. The study findings shall not be generalized at the national-level as the data were collected from one district only. Since SRH was a self-reported outcome, it might be affected by social bias and conformity.

## Supplementary Information

**Additional file 1.** Structured schedule for adolescent girls.

**Additional file 2.** Structured schedule for mothers.

## Data Availability

The datasets generated and/or analysed during the current study are not publicly available as this data is a part of corresponding author’s PhD research work and it was collected by corresponding author to receive the PhD degree, but are available from the corresponding author on reasonable request.

## Abbreviations

SC/ST: Scheduled castes/scheduled tribes
OBC: Other backward class
SRH: Self-rated health
