# Supplementary material for: How do responses vary between mothers and their daughters on measuring daughter’s self-rated health (SRH): a study among school-going adolescent girls in the primary setting of Varanasi, India
Source: BMC Res Notes. 2022 Sep 5;15:289. doi: 10.1186/s13104-022-06174-1 (PMC9446715; doi:10.1186/s13104-022-06174-1)
Supplement: Supplementary file 1 — Additional file 1. Structured schedule for adolescent girls. [file 13104_2022_6174_MOESM1_ESM.pdf]

**Social Capital and its Association with Health, Wellbeing and Educational Aspirations of Adolescent School-going Girls: A Study of Varanasi City**

SCHEDULE NO: ☐ ☐ ☐

CONFIDENTIAL

For Research Purpose Only

( स्थापना / Established in 1956)

बेहतर भविष्य के लिए क्षमता निर्माण

Capacity Building for a Better Future

# Questionnaire for Adolescent Girl

| IDENTIFICATION | |
|---|---|
| WARD NO: | NAME OF RESPONDENT: |
| WARD NAME: | DATE: |
| SCHOOL NAME: | |

| Interview Status | |
|---|---|
| Completed ………… 1 | |
| Incomplete ………… 2 | Refused ……………………. 3 |

CONFIDENTIAL

For Research Purpose Only

# Informed Consent Form for Principal

## Social Capital and its Association with Health, Wellbeing and Educational Aspirations of Adolescent School-going Girls: A Study of Varanasi City

---

**International Institute for Population Sciences (IIPS)**
**Deemed University**
**(Ministry of Health and Family Welfare, Govt. of India)**
**Deonar, Mumbai-400088**

---

Namaste, My name is Ratna Patel. I am pursuing my PhD at the International Institute for Population Sciences, Mumbai. My Ph. D. research topic is "Social Capital and its Association with Health, Wellbeing and Educational Aspirations of Adolescent School-going Girls: A Study of Varanasi City". In this study, I am exploring the role of school, family, and neighbourhood for the overall well-being of adolescent girls. The study will be useful in understanding the important roles played by agencies like school, family, and neighbourhood. I will be giving questionnaire to the girl students from your school studying in class $8^{th}$ to $12^{th}$. They will after getting consent from their parents will fill it and return it to me .I will be asking questions related to school, family, and neighbourhood. The questions usually take 20-30 minutes.

I assure you that complete privacy will be maintained and true identity of the students will remain anonymous in research.Participation in this research is voluntary and the information collected will be kept confidential. The students can discontinue the interview at any point and choose not to answer the questions they don't like.
If you have any question about the survey, you can ask me. I would be glad to answer your queries. For more information, you can also contact to my supervisor at International Institute for Population Sciences, Mumbai.

Dr.Dhananjay W, Bansod
Associate Professor
Department of Public Health and Mortality Studies
International Institute for Population Sciences
Mumbai- 400088
dhananjay@iips.net

Phone: 022-42372496

Ratna Patel
Contact Number: 9769330105

May I begin the interview now? Yes/No

Do you agree to participate in this study?

Agreed with Signature ………… 1 } Start
Agreed without Signature ……….. 2 }
Not Agreed ……………………… 3 → End

Signature of the interviewer:
Date:

CONFIDENTIAL

For Research Purpose Only

# Informed Consent Form for Principal

## Social Capital and its Association with Health, Wellbeing and Educational Aspirations of Adolescent School-going Girls: A Study of Varanasi City

**International Institute for Population Sciences (IIPS)**
**Deemed University**
**(Ministry of Health and Family Welfare, Govt. of India)**
**Deonar, Mumbai-400088**

---

नमस्ते, मेरा नाम रत्ना पटेल है। मैं इंटरनेशनल इंस्टीट्यूट फॉर पॉपुलेशन साइंसेज, मुंबई में पीएचडी कर रही हूं। मेरा पीएचडी शोध विषय "सोशल कैपिटल एंड इट्स एसोसिएशन विद हेल्थ, वेलबीइंग एंड एजुकेशनल एस्पिरेशंस ऑफ एडोल्सेंट स्कूल-गोइंग गर्ल्स: अ स्टडी ऑफ वाराणसी सिटी" है। इस अध्ययन में, मैं किशोरी लड़कियों की समग्र हित के लिए स्कूल, परिवार और पड़ोस की भूमिका तलाश रही हूं। यह अध्ययन स्कूल, परिवार और पड़ोस जैसी एजेंसियों द्वारा निभाई गई महत्वपूर्ण भूमिकाओं को समझने में उपयोगी होगा।मैं आपके विद्यालय की 8 वीं से 12 वीं कक्षा में पढ़ने वाली छात्राओं को प्रश्नावली देरही हूँ।वे अपने माता-पिता से सहमति प्राप्त करने के बाद इसे भर देंगे और इसे मुझे वापस कर देंगे मैं स्कूल, परिवार और पड़ोस से संबंधित प्रश्न करूँगी। प्रश्न करने में आमतौर पर 25-30 मिनट लगते हैं।

मैं आपको विश्वास दिलाती हूँ कि पूर्ण गोपनीयता बनाए रखी जाएगी और छात्रों की सही पहचान अनुसंधान में गुमनाम रहेगी। इस शोध में भागीदारी स्वैच्छिक है और एकत्र की गई जानकारी को गोपनीय रखा जाएगा। आप किसी भी बिंदु पर साक्षात्कार को बंद कर सकते हैं और उन सवालों के जवाब नहीं देने के लिए चुन सकते हैं जो आपको पसंद नहीं हैं। यदि आपके पास सर्वेक्षण के बारे में कोई प्रश्न है, तो आप मुझसे पूछ सकते हैं। मुझे आपके प्रश्नों का उत्तर देने में खुशी होगी। अधिक जानकारी के लिए, आप इंटरनेशनल इंस्टीट्यूट फॉर पॉपुलेशन साइंसेज, मुंबई में मेरे पर्यवेक्षक से भी संपर्क कर सकते हैं।

डॉ.धनंजय डब्ल्यू, बनसोड
एसोसिएट प्रोफेसर
सार्वजनिक स्वास्थ्य और मृत्यु दर विभाग
जनसंख्या विज्ञान के लिए अंतर्राष्ट्रीय संस्थान
मुंबई- 400088

dhananjay@iips.net

Phone: 022-42372496

रत्ना पटेल
संपर्क नंबर: 9769330105

क्या आप इस अध्ययन में भाग लेने के लिए सहमत हैं? हाँ/नहीं

हस्ताक्षर के साथ सहमत ........... 1 ⎫ प्रारंभ
हस्ताक्षर के बिना सहमत .......... 2 ⎭

सहमत नहीं .............................. 3 → अंत

साक्षात्कारकर्ता के हस्ताक्षर:

दिनांक:

CONFIDENTIAL

For Research Purpose Only

# Informed Consent Form for Mother

## Social Capital and its Association with Health, Wellbeing and Educational Aspirations of Adolescent School-going Girls: A Study of Varanasi City

**International Institute for Population Sciences (IIPS)**
**Deemed University**
**(Ministry of Health and Family Welfare, Govt. of India)**
**Deonar, Mumbai-400088**

--------------------------------------------------------------------------------------------------------------------

Namaste, My name is Ratna Patel. I am pursuing my PhD at the International Institute for Population Sciences, Mumbai. My Ph. D. research topic is "Social Capital and its Association with Health, Wellbeing and Educational Aspirations of Adolescent School-going Girls: A Study of Varanasi City". In this study, I am exploring the role of school, family, and neighbourhood for the overall well-being of adolescent girls. The study will be useful in understanding the important roles played by agencies like school, family, and neighbourhood.

I will be giving questionnaire to your daughter. After getting your consent she will fill it and return it to me.

The questionnaire has questions are related to her school, family, and neighbourhood. The questions usually take 20-30 minutes.

I assure you that complete privacy will be maintained and true identity of your daughter will remain anonymous in research. Participation in this research is voluntary and the information collected will be kept confidential. Your daughter can discontinue the interview at any point and choose not to answer the questions they don't like.

If you have any question about the survey, you can ask me. I would be glad to answer your queries. For more information, you can also contact to my supervisor at International Institute for Population Sciences, Mumbai.

Dr.Dhananjay W, Bansod
Associate Professor
Department of Public Health and Mortality Studies
International Institute for Population Sciences
Mumbai- 400088
dhananjay@iips.net

Phone: 022-42372496

Ratna Patel
Contact Number: 9769330105

May I begin the interview now? Yes/No

Do you agree to participate in this study?

Agreed with Signature ………… 1 } Start
Agreed without Signature ……….. 2
Not Agreed ……………………… 3 → End

Signature of the interviewer:
Date:

CONFIDENTIAL

For Research Purpose Only

# Informed Consent Form for Mother

## Social Capital and its Association with Health, Wellbeing and Educational Aspirations of Adolescent School-going Girls: A Study of Varanasi City

**International Institute for Population Sciences (IIPS)**
**Deemed University**
**(Ministry of Health and Family Welfare, Govt. of India)**
**Deonar, Mumbai-400088**

-----------------------------------------------------------------------------------------------------------------------------

नमस्ते, मेरा नाम रत्ना पटेल है। मैं इंटरनेशनल इंस्टीट्यूट फॉर पॉपुलेशन साइंसेज, मुंबई में पीएचडी कर रही हूं। मेरा पीएचडी शोध विषय "सोशल कैपिटल एंड इट्स एसोसिएशन विद हेल्थ, वेलबीइंग एंड एजुकेशनल एस्पिरेशंस ऑफ एडोल्सेंट स्कूल-गोइंग गर्ल्स: अ स्टडी ऑफ वाराणसी सिटी" है। इस अध्ययन में, मैं किशोरी लड़कियों की समग्र हित के लिए स्कूल, परिवार और पड़ोस की भूमिका तलाश रही हूं। यह अध्ययन स्कूल, परिवार और पड़ोस जैसी एजेंसियों द्वारा निभाई गई महत्वपूर्ण भूमिकाओं को समझने में उपयोगी होगा।मैं आपकी बेटी को प्रश्नावली दे रही हूँ ।आपकी सहमति मिलने के बाद वह इसे भरेगी और मुझे लौटा देगी। मैं स्कूल, परिवार और पड़ोस से संबंधित प्रश्न करूँगी। प्रश्न करने में आमतौर पर 25-30 मिनट लगते हैं।

मैं आपको विश्वास दिलाती हूँ कि पूर्ण गोपनीयता बनाए रखी जाएगी और आपकी बेटी की सही पहचान अनुसंधान में गुमनाम रहेगी। इस शोध में भागीदारी स्वैच्छिक है और एकत्र की गई जानकारी को गोपनीय रखा जाएगा। आपकी बेटी किसी भी बिंदु पर साक्षात्कार को बंद कर सकती हैं और उन सवालों के जवाब नहीं देने के लिए चुन सकती हैं जो उसे पसंद नहीं हैं। यदि आपके पास सर्वेक्षण के बारे में कोई प्रश्न है, तो आप मुझसे पूछ सकते हैं। मुझे आपके प्रश्नों का उत्तर देने में खुशी होगी। अधिक जानकारी के लिए, आप इंटरनेशनल इंस्टीट्यूट फॉर पॉपुलेशन साइंसेज, मुंबई में मेरे पर्यवेक्षक से भी संपर्क कर सकते हैं।

डॉ.धनंजय डब्ल्यू, बनसोड
एसोसिएट प्रोफेसर
सार्वजनिक स्वास्थ्य और मृत्यु दर विभाग
जनसंख्या विज्ञान के लिए अंतर्राष्ट्रीय संस्थान
मुंबई- 400088

dhananjay@iips.net

Phone: 022-42372496

रत्ना पटेल
संपर्क नंबर: 9769330105

क्या आप इस अध्ययन में भाग लेने के लिए सहमत हैं? हाँ/नहीं

हस्ताक्षर के साथ सहमत ........... 1 } प्रारंभ

हस्ताक्षर के बिना सहमत .......... 2 }

सहमत नहीं ............................. 3 → अंत

साक्षात्कारकर्ता के हस्ताक्षर:

दिनांक:

**SECTION I: PERSONAL INFORMATION:**

| S. No. | Questions | Coding Categories | | Skip/ Go to |
|---|---|---|---|---|
| 1. | What is your date of birth? | 1.A Day of Birth..........\|___\|___\|<br>1.B Month of birth .....\|___\|___\|<br>1.C Year of Birth ........\|___\|___\|___\|___\|<br>Don't Know.......................................98 | | |
| 2. | How old were you on your last birthday?<br>Compare and correct 1 and 2, if inconsistent | Age in completed years........\|___\|___\| | | |
| 3. | In which class are you studying? | 8th............................................ ..<br>9th...............................................<br>10th ...........................................<br>11th ...........................................<br>12th ........................................... | 1<br>2<br>3<br>4<br>5 | |
| 4. | Years of schooling | 7 to 15 years....................\|___\|___\| | | |
| 5. | How many brothers and sisters you have? | Brothers ....................\|___\|___\|<br>Sisters .......................\|___\|___\|<br>No sibling..................................... | <br><br>98 → | <br>Skip<br>to 9 |
| 6. | Are you the eldest of all of your siblings? | Yes...............................................<br>No............................................... | 1<br>2 | |
| 7. | How many of your siblings are elder to you?<br>**(Do not answer if you are the eldest)** | Brother …………………..<br>Sister ……………………. | | |
| 8. | Educational Status of each of your siblings | S. No. Brother/Sister Class<br>1<br>2<br>3<br>4<br>5<br>6 | | |
| **Mass Media Exposure** | | | | |
| 9. | Do you read a newspaper or magazine? | Almost every day .........................<br>At least once a week ...................<br>Not at all ................................... | 1<br>2<br>3 | |
| 10. | Do you listen to radio? | Almost every day .........................<br>At least once a week .....................<br>Not at all .................................... | 1<br>2<br>3 | |
| 11. | Do you watch television? | Almost every day .......................<br>At least once a week ..................<br>Not at all .. | 1<br>2<br>3 | |
| 12. | Do you go to movies/theatre? | At least once a week .....................<br>At least once a month ..................<br>Not at all ...................................... | 1<br>2<br>3 | |
| 13. | Do you own a mobile? | Yes .............................................<br>No ............................................... | 1<br>2 | |
| 14. | Are you active on social media platforms? | Yes ...............................................<br>No ............................................... | 1<br>2 | |
| 15. | How much time do you spend on social media per day? | Less than one hour ......................<br>More than one hour ..................... | 1<br>2 | |
| 16. | Name of the school | .....................................................<br>..................................................... | | |

| | | | |
|---|---|---|---|
| 17. | Type of school | Government ................................. 1<br>Private ......................................... 2<br>Trust ............................................ 3<br>Municipal corporation ................ 4<br>Religious ..................................... 5 | |
| 18. | Nature of the school | Girls only ................................... 1<br>Co-education ............................. 2 | |
| 19. | Medium taught in the school | Hindi .......................................... 1<br>English ....................................... 2<br>Other Language ........................... 3 | |
| 20. | Syllabus followed | State ........................................... 1<br>CBSE .......................................... 2<br>ICSE ........................................... 3<br>Any Other.................................... 98 | |
| 21. | Religion | Hindu .......................................... 1<br>Muslim ........................................ 2<br>Christian ...................................... 3<br>Other religion .............................. 4 | |
| 22. | Mother's education | Illiterate ...................................... 1<br>Literate but no education ............. 2<br>Primary ........................................ 3<br>Secondary.................................... 4<br>Higher ......................................... 5 | |
| 23. | Father's education | Illiterate ...................................... 1<br>Literate but no education ............. 2<br>Primary ........................................ 3<br>Secondary .................................... 4<br>Higher ......................................... 5 | |
| 24. | Occupation of Mother | Government job........................... 1<br>Private job ................................... 2<br>Self-employed ............................ 3<br>House-wife .................................. 4<br>Other (Specify)...........................<br>.................................................... 98 | |
| 25. | Occupation of father | Government job ........................... 1<br>Private job ................................... 2<br>Self-employed ............................. 3<br>No job ......................................... 4<br>Other (Specify)...........................<br>.................................................... 98 | |
| 26. | Information regarding working parents | One working at distance place ..... 1<br>Both working at distance place .... 2<br>One working at nearby distance ... 3<br>Both working at nearby ............... 4 | |
| 27 | Which type of family are you coming from? | Nuclear Family ........................ 1<br>Joint Family .............................. 2<br>Extended family........................ 3 | |

**SECTION II: SOCIAL CAPITAL VARIABLES**

| S. No. | Questions | Coding Categories | Skip/ Go to |
|---|---|---|---|
| **FAMILY SOCIAL CAPITAL** | | | |
| 28 | Do you feel that your family understand and give attention to you? | Strongly Agree ...................... 1<br>Agree ..................................... 2<br>Neither Agree nor Disagree .... 3<br>Disagree .................................. 4<br>Strongly Disagree .................. 5 | |

| | | | | |
|---|---|---|---|---|
| 29 | When you return from your school, who generally receive you at home? | Any of the parents ....................<br>Grandparents ...........................<br>Any of the realtive .................<br>No one .................................. | 1<br>2<br>3<br>4 | |
| 30 | Everyone in my family loves me and understands me | Strongly Agree ......................<br>Agree .....................................<br>Neither Agree nor Disagree .....<br>Disagree .................................<br>Strongly Disagree.................... | 1<br>2<br>3<br>4<br>5 | |
| 31 | In my family, we talk about important things | Strongly Agree ......................<br>Agree .....................................<br>Neither Agree nor Disagree .....<br>Disagree .................................<br>Strongly Disagree.................... | 1<br>2<br>3<br>4<br>5 | |
| 32 | In my family, we listen to each other | Strongly Agree ......................<br>Agree .....................................<br>Neither Agree nor Disagree .....<br>Disagree .................................<br>Strongly Disagree.................... | 1<br>2<br>3<br>4<br>5 | |
| 33 | In my family, we support each other | Strongly Agree ......................<br>Agree .....................................<br>Neither Agree nor Disagree .....<br>Disagree .................................<br>Strongly Disagree.................... | 1<br>2<br>3<br>4<br>5 | |
| 34 | In my family, we resolve misunderstanding | Strongly Agree ......................<br>Agree .....................................<br>Neither Agree nor Disagree .....<br>Disagree .................................<br>Strongly Disagree.................... | 1<br>2<br>3<br>4<br>5 | |
| 35 | In my family, we have dinner together | Strongly Agree ......................<br>Agree .....................................<br>Neither Agree nor Disagree .....<br>Disagree .................................<br>Strongly Disagree.................... | 1<br>2<br>3<br>4<br>5 | |
| 36 | In my family, we generally go out for movies/picnic/dinner together on a regular basis | Strongly Agree ......................<br>Agree .....................................<br>Neither Agree nor Disagree .....<br>Disagree .................................<br>Strongly Disagree.................... | 1<br>2<br>3<br>4<br>5 | |
| **NEIGHBOURHOOD SOCIAL CAPITAL** | | | | |
| 37 | Do you feel people trust each other in your neighbourhood? | Strongly Agree ......................<br>Agree .....................................<br>Neither Agree nor Disagree .....<br>Disagree .................................<br>Strongly Disagree.................... | 1<br>2<br>3<br>4<br>5 | |
| 38 | Do you feel that your neighbours step in to criticise someone’s deviant behaviour during school? | Strongly Agree ......................<br>Agree .....................................<br>Neither Agree nor Disagree .....<br>Disagree .................................<br>Strongly Disagree.................... | 1<br>2<br>3<br>4<br>5 | |
| 39 | In my neighbourhood, I feel safe | Strongly Agree ......................<br>Agree .....................................<br>Neither Agree nor Disagree .....<br>Disagree .................................<br>Strongly Disagree.................... | 1<br>2<br>3<br>4<br>5 | |
| **SCHOOL SOCIAL CAPITAL** | | | | |
| 40 | Do you feel teachers and students trust each other in your school? (Vertical School Trust) | Strongly Agree ......................<br>Agree ..................................... | 1<br>2 | |

| | | | | |
|---|---|---|---|---|
| | | Neither Agree nor Disagree .....<br>Disagree .................................<br>Strongly Disagree.................... | 3<br>4<br>5 | |
| 41 | Do you feel students trust each other in your school? (Horizontal School Trust) | Strongly Agree ......................<br>Agree .....................................<br>Neither Agree nor Disagree .....<br>Disagree .................................<br>Strongly Disagree.................... | 1<br>2<br>3<br>4<br>5 | |
| 42 | Do you feel student collaborate with each other in your school? (Reciprocity at school) | Strongly Agree ......................<br>Agree .....................................<br>Neither Agree nor Disagree .....<br>Disagree .................................<br>Strongly Disagree.................... | 1<br>2<br>3<br>4<br>5 | |
| 43 | I feel that my teachers appreciate me | Strongly Agree ......................<br>Agree .....................................<br>Neither Agree nor Disagree .....<br>Disagree .................................<br>Strongly Disagree.................... | 1<br>2<br>3<br>4<br>5 | |
| 44 | I feel that our teachers treat students fairly | Strongly Agree ......................<br>Agree .....................................<br>Neither Agree nor Disagree .....<br>Disagree .................................<br>Strongly Disagree.................... | 1<br>2<br>3<br>4<br>5 | |
| 45 | I feel that my teacher understand my problems | Strongly Agree ......................<br>Agree .....................................<br>Neither Agree nor Disagree .....<br>Disagree .................................<br>Strongly Disagree.................... | 1<br>2<br>3<br>4<br>5 | |
| 46 | I feel our teachers acknowledge and respect student's own opinion | Strongly Agree ......................<br>Agree .....................................<br>Neither Agree nor Disagree .....<br>Disagree .................................<br>Strongly Disagree.................... | 1<br>2<br>3<br>4<br>5 | |
| 47 | I usually get along well with my teachers | Strongly Agree ......................<br>Agree .....................................<br>Neither Agree nor Disagree .....<br>Disagree .................................<br>Strongly Disagree.................... | 1<br>2<br>3<br>4<br>5 | |
| 48 | How do you feel about school at present | Excellent .................................<br>Good.........................................<br>Fair..........................................<br>Poor.........................................<br>Very poor.................................<br>Other (specify) | 1<br>2<br>3<br>4<br>5<br>98 | |
| **Relationship with classmates using three items** | | | | |
| 49 | Students enjoy being together | Strongly Agree ......................<br>Agree .....................................<br>Neither Agree nor Disagree .....<br>Disagree .................................<br>Strongly Disagree.................... | 1<br>2<br>3<br>4<br>5 | |
| 50 | Most students are kind and helpful | Strongly Agree ......................<br>Agree .....................................<br>Neither Agree nor Disagree .....<br>Disagree .................................<br>Strongly Disagree.................... | 1<br>2<br>3<br>4<br>5 | |
| 51 | Students accept me as I am | Strongly Agree ......................<br>Agree .....................................<br>Neither Agree nor Disagree .....<br>Disagree ................................ | 1<br>2<br>3<br>4 | |

| | | | | |
|---|---|---|---|---|
| | | Strongly Disagree.................... | 5 | |
| **Students- Teachers Relationship using three items scale** | | | | |
| 52 | Teachers accept me as I am | Strongly Agree ......................<br>Agree .....................................<br>Neither Agree nor Disagree .....<br>Disagree .................................<br>Strongly Disagree.................... | 1<br>2<br>3<br>4<br>5 | |
| 53 | Teachers care about me | Strongly Agree ......................<br>Agree .....................................<br>Neither Agree nor Disagree .....<br>Disagree .................................<br>Strongly Disagree.................... | 1<br>2<br>3<br>4<br>5 | |
| 54 | I feel a lot of trust in my teachers | Strongly Agree ......................<br>Agree .....................................<br>Neither Agree nor Disagree .....<br>Disagree .................................<br>Strongly Disagree.................... | 1<br>2<br>3<br>4<br>5 | |
| 55 | State the facilities in your school | Adequate teaching Staff ............<br>Proper Class room .....................<br>Availability of basic amenities...<br>(drinking water, washrooms)<br>Playground.................................<br>Playing equipments ...................<br>Library ....................................<br>Lab Facilities ...........................<br>Good school environment Extra<br>Curricular Activities ................ | 1<br>2<br>3<br><br>4<br>5<br>6<br>7<br>8<br>9 | |
| 56 | Do you satisfied with the facilities available at your school? | Yes ......................................<br>No ....................................... | 1<br>2 | |

**SECTION *III*: HEALTH, WELL-BEING AND SOCIAL CAPITAL**

| S. No. | Questions | Coding Categories | | Skip/ Go to |
|---|---|---|---|---|
| 57 | Do you think that your parents feel worried when you get ill? | Yes ..................................<br>No .................................... | 1<br>2 | |
| 58 | Do you think that your other siblings get better treatment when they fell ill? | Yes ..................................<br>No .................................... | 1<br>2 | |
| 59 | Do you have any hospital in your nearby area? | Yes ..................................<br>No .................................... | 1<br>2 → | Skip to 61 |
| 60 | If yes, type of hospital | Private Hospital ................<br>Public Hospital .................<br>Medical Collage................<br>Clinics...............................<br>Charity run Hospital .......... | 1<br>2<br>3<br>4<br>5 | |
| 61 | When you fell ill, your parents prefer to take you to which type of hospital? | Private Hospital ................<br>Public Hospital .................<br>Medical Collage................<br>Clinics...............................<br>Charity run Hospital ..........<br>Medicine from shop...........<br>Other (specify)_________<br>Did Nothing...................... | 1<br>2<br>3<br>4<br>5<br>6<br>96<br>98 | |
| 62 | The hospital you visit, when you fell ill, is generally how far from your home? | Within 5 km .....................<br>Within 10 km ..................<br>Within 15 km ..................<br>Within 20 km ..................<br>Within 25 km ..................<br>More than 25 km ............. | 1<br>2<br>3<br>4<br>5<br>6 | |

| | | | |
|---|---|---|---|
| | | Don't know 98 | |
| 63 | The hospital your other siblings visit, when they fell ill, is generally how far from your home? | Within 5 km ...................... 1<br>Within 10 km .................. 2<br>Within 15 km .................. 3<br>Within 20 km .................. 4<br>Within 25 km .................. 5<br>More than 25 km ............. 6<br>Don't know...................... 98 | |
| 64 | Do you think that in your neighbourhood there is no any good hospital? | Yes .................................. 1<br>No .................................... 2 | |
| 65 | Do you know any good hospital which is not very near to your home? | Yes .................................. 1<br>No .................................... 2 | |
| 66 | Did you ever fell seriously ill? | Yes .................................. 1<br>No .................................... 2 | |
| 67 | Have you ever visited that good hospital when you fell seriously ill? | Yes .................................. 1<br>No .................................... 2 | |
| 68 | Do your other siblings visited that good hospital when they fell seriously ill? | Yes .................................. 1<br>No .................................... 2 | |
| 69 | Do the boys in your home visit better hospital (in terms of facilities) than girls at your home? | Yes .................................. 1<br>No .................................... 2 | |
| 70 | Your neighbours show concern when you fell ill? | Yes .................................. 1<br>No .................................... 2 | |
| 71 | Do the people from your neighbourhood come to meet you when you fell ill? | Yes .................................. 1<br>No .................................... 2 | |
| 72 | Will your neighbours lend your parents some money, if they need to cover medical expenses? | Yes .................................. 1<br>No .................................... 2 | |
| 73 | Do you think that your school has got the basic medical facilities covered? | Yes .................................. 1<br>No ..................................... 2 | |
| 74 | Do you feel that your school authorities show prompt response when you do not feel good in terms of your health? | Yes .................................. 1<br>No .................................... 2 | |
| 75 | Does school authority understood your health concern and act accordingly? | Yes .................................. 1<br>No .................................... 2 | |

## SECTION IV: EDUCATIONAL OUTCOMES, EDUCATIONAL ASPIRATIONS, FUTURE GOALS, AND SOCIAL CAPITAL

| S. No. | Questions | Coding Categories | Skip/ Go to |
|---|---|---|---|
| 76 | Do you want to pursue higher study after school? | Yes ................................ 1<br>No .................................. 2 | If no, skip the section |
| 77 | Will your parents allow you to pursue higher study? | Yes ................................ 1<br>No .................................. 2 | |
| 78 | Are your parents supportive for your study? | Yes ................................ 1<br>No .................................. 2 | |
| 79 | Does your parents have high hopes on you for your educational achievements? | Yes ................................ 1<br>No .................................. 2 | |
| 80 | Do you feel pressure of achievement in study because of your parents? | Yes ................................ 1<br>No .................................. 2 | |
| 81 | Does your school teachers motivate you for you good performance in school? | Yes ................................ 1<br>No .................................. 2 | |
| 82 | Do you have a future goal in your life? | Yes ................................ 1<br>No .................................. 2 → | If no, skip to 86 |
| 83 | Have you ever discussed your future goal with your parents? | Yes ................................ 1<br>No .................................. 2 | |
| 84 | Does your parents are supportive for your future goal? | Yes ................................ 1 →<br>No .................................. 2 | If yes, skip 86 |
| 85 | Why parents are not supportive for your future | ....................................... | |

| | | | |
|---|---|---|---|
| | goal? Expain | ....................................... | |
| 86 | Does your teachers understand your educational capacity and act accordingly to that? | Yes ................................ 1<br>No .................................. 2 | |

**SECTION V: GIRL'S AUTONOMY AND SOCIAL CAPITAL**

| S. No. | Questions | Coding Categories | Skip/ Go to |
|---|---|---|---|
| 87 | Do you say that you enjoy personal space at home? | Yes ................................ 1<br>No .................................. 2 | |
| 88 | Are you free to choose which stream you will choose after your intermediate? | Yes ................................ 1<br>No .................................. 2 | |
| 89 | Do you feel that your brothers are enjoying more freedom than you? | Yes ................................ 1<br>No .................................. 2 | |
| 90 | Your parents try to impose their wishes on you for your academic decisions? | Yes ................................ 1<br>No .................................. 2 | |
| 91 | Do you go for tuition? | Yes ................................ 1<br>No ................................. 2 → | Skip to 93 |
| 92 | If yes, who decides the tutor? | Self ................................ 1<br>Parents ............................. 2<br>You along with parents .... 3<br>Your neighbours .............. 4<br>Others (specify)________ 98 | |
| 93 | Does tutor comes to your home or you go to his/her place for tuition? | Tutor comes to home ....... 1<br>I go to tutor's home ........ 2<br>I go to tutor's coaching..... 3<br>centre | |
| 94 | Are you free to decide your Academic decisions? | Yes ................................ 1<br>No .................................. 2 | |
| 95 | Do anyone stop you for roaming around your neighbourhood as per your wishes? | Yes ................................ 1<br>No .................................. 2 | |

**SECTION VI: KIDCREEN 52 QUESTIONNAIRE**

| **SECTION 1: Physical activities and Health** | | |
|---|---|---|
| 96 | In General, How would you say your health is? | Excellent ...................... 1<br>Very Good ................. 2<br>Good ............................ 3<br>Fair ............................ 4<br>Poor ........................... 5 |
| 97 | Thinking about last week:<br>Have you felt physically fit and well? | Not at all..................... 1<br>Slightly......................... 2<br>Moderately.................. 3<br>Very.............................. 4<br>Extremely................... 5 |
| 98 | Thinking about last week:<br>Have you been physically active (e.g. running, climbing, cycling)? | Not at all..................... 1<br>Slightly......................... 2<br>Moderately.................. 3<br>Very.............................. 4<br>Extremely................... 5 |
| 99 | Thinking about last week:<br>Have you been able to run well? | Not at all..................... 1<br>Slightly......................... 2<br>Moderately.................. 3 |

| | | |
|---|---|---|
| | | Very............................. 4<br>Extremely................... 5 |
| 100 | Thinking about last week:<br>Have you felt full of energy? | Never.......................... 1<br>Almost never.............. 2<br>Sometimes................. 3<br>Almost always............ 4<br>Always....................... 5 |
| **Section 2: Feelings** | | |
| 101 | Thinking about last week:<br>Have your life been enjoyable? | Not at all..................... 1<br>Slightly........................ 2<br>Moderately.................. 3<br>Very............................. 4<br>Extremely................... 5 |
| 102 | Thinking about last week:<br>Have you felt pleased that you are alive? | Not at all..................... 1<br>Slightly........................ 2<br>Moderately.................. 3<br>Very............................. 4<br>Extremely................... 5 |
| 103 | Thinking about last week:<br>Have you felt satisfies with your life? | Not at all..................... 1<br>Slightly........................ 2<br>Moderately.................. 3<br>Very............................. 4<br>Extremely................... 5 |
| 104 | Thinking about last week:<br>Have you been in good mood? | Never.......................... 1<br>Almost never.............. 2<br>Sometimes................. 3<br>Almost always............ 4<br>Always....................... 5 |
| 105 | Thinking about last week:<br>Have you felt Cheerful? | Never.......................... 1<br>Almost never.............. 2<br>Sometimes................. 3<br>Almost always............ 4<br>Always....................... 5 |
| 106 | Thinking about last week:<br>Have you had fun? | Never.......................... 1<br>Almost never.............. 2<br>Sometimes................. 3<br>Almost always............ 4<br>Always....................... 5 |
| **Section 3: General Mood** | | |
| 107 | Thinking about last week:<br>Have you felt that you do everything badly? | Never.......................... 1<br>Almost never.............. 2<br>Sometimes................. 3<br>Almost always............ 4<br>Always....................... 5 |
| 108 | Thinking about last week:<br>Have you felt sad? | Never.......................... 1<br>Almost never.............. 2<br>Sometimes................. 3<br>Almost always............ 4<br>Always....................... 5 |
| 109 | Thinking about last week:<br>Have you felt so bad that you didn’t want to do anything? | Never.......................... 1<br>Almost never.............. 2<br>Sometimes................. 3<br>Almost always............ 4 |

| | | |
|---|---|---|
| | | Always....................... 5 |
| 110 | Thinking about last week:<br>Have you felt that everything in your life goes wrong? | Never.......................... 1<br>Almost never.............. 2<br>Sometimes................. 3<br>Almost always............ 4<br>Always....................... 5 |
| 111 | Thinking about last week:<br>Have you felt fed up? | Never.......................... 1<br>Almost never.............. 2<br>Sometimes................. 3<br>Almost always............ 4<br>Always....................... 5 |
| 112 | Thinking about last week:<br>Have you felt lonely? | Never.......................... 1<br>Almost never.............. 2<br>Sometimes................. 3<br>Almost always............ 4<br>Always....................... 5 |
| 113 | Thinking about last week:<br>Have you felt under pressure? | Never.......................... 1<br>Almost never.............. 2<br>Sometimes................. 3<br>Almost always............ 4<br>Always....................... 5 |
| **Section 4: About Yourself** | | |
| 114 | Thinking about last week:<br>Have you been happy with the way you are? | Never.......................... 1<br>Almost never.............. 2<br>Sometimes................. 3<br>Almost always............ 4<br>Always....................... 5 |
| 115 | Thinking about last week:<br>Have you been happy with your clothes? | Never.......................... 1<br>Almost never.............. 2<br>Sometimes................. 3<br>Almost always............ 4<br>Always....................... 5 |
| 116 | Thinking about last week:<br>Have you been worried about the way you look? | Never.......................... 1<br>Almost never.............. 2<br>Sometimes................. 3<br>Almost always............ 4<br>Always....................... 5 |
| 117 | Thinking about last week:<br>Have you felt jealous of the way other girls look? | Never.......................... 1<br>Almost never.............. 2<br>Sometimes................. 3<br>Almost always............ 4<br>Always....................... 5 |
| 118 | Thinking about last week:<br>Would you like to change something about your body? | Never.......................... 1<br>Almost never.............. 2<br>Sometimes................. 3<br>Almost always............ 4<br>Always....................... 5 |
| **Section 5: Free Time** | | |
| 119 | Thinking about last week:<br>Have you had enough time for yourself? | Never.......................... 1<br>Almost never.............. 2<br>Sometimes................. 3<br>Almost always............ 4<br>Always....................... 5 |

| | | |
|---|---|---|
| 120 | Thinking about last week:<br>Have you been able to do the things that you want to do in your free time? | Never.......................... 1<br>Almost never.............. 2<br>Sometimes................. 3<br>Almost always............ 4<br>Always....................... 5 |
| 121 | Thinking about last week:<br>Have you had enough opportunity to be outside? | Never.......................... 1<br>Almost never.............. 2<br>Sometimes................. 3<br>Almost always............ 4<br>Always....................... 5 |
| 122 | Thinking about last week:<br>Have you had enough time to meet friends? | Never.......................... 1<br>Almost never.............. 2<br>Sometimes................. 3<br>Almost always............ 4<br>Always....................... 5 |
| 123 | Thinking about last week:<br>Have you been able to choose what to do in your free time? | Never.......................... 1<br>Almost never.............. 2<br>Sometimes................. 3<br>Almost always............ 4<br>Always....................... 5 |
| **Section 6: Family and Home Life** | | |
| 124 | Thinking about last week:<br>Have your parents understood you? | Not at all..................... 1<br>Slightly........................ 2<br>Moderately.................. 3<br>Very............................. 4<br>Extremely................... 5 |
| 125 | Thinking about last week:<br>Have you felt loved by your parents? | Not at all..................... 1<br>Slightly........................ 2<br>Moderately.................. 3<br>Very............................. 4<br>Extremely................... 5 |
| 126 | Thinking about last week:<br>Have you been happy at home? | Never.......................... 1<br>Almost never.............. 2<br>Sometimes................. 3<br>Almost always............ 4<br>Always....................... 5 |
| 127 | Thinking about last week:<br>Have your parents had enough time for you? | Never.......................... 1<br>Almost never.............. 2<br>Sometimes................. 3<br>Almost always............ 4<br>Always....................... 5 |
| 128 | Thinking about last week:<br>Have your parents treated you fairly? | Never.......................... 1<br>Almost never.............. 2<br>Sometimes................. 3<br>Almost always............ 4<br>Always....................... 5 |
| 129 | Thinking about last week:<br>Have you been able to talk to your parents when you wanted to? | Never.......................... 1<br>Almost never.............. 2<br>Sometimes................. 3<br>Almost always............ 4<br>Always....................... 5 |
| **Section 7: Money Matters** | | |
| 130 | Thinking about last week: | Never.......................... 1 |

| | | | |
|---|---|---|---|
| | Have you had enough money to do the same things as your friends do? | Almost never..............<br>Sometimes.................<br>Almost always............<br>Always....................... | 2<br>3<br>4<br>5 |
| 131 | Thinking about last week:<br>Have you had enough money for your expenses? | Never..........................<br>Almost never..............<br>Sometimes.................<br>Almost always............<br>Always....................... | 1<br>2<br>3<br>4<br>5 |
| 132 | Thinking about last week:<br>Do you have enough money to do things with your friends? | Not at all.....................<br>Slightly.........................<br>Moderately..................<br>Very.............................<br>Extremely................... | 1<br>2<br>3<br>4<br>5 |
| **Section 8: Friends** | | | |
| 133 | Thinking about last week:<br>Have you spent time with your friends? | Never..........................<br>Almost never..............<br>Sometimes.................<br>Almost always............<br>Always....................... | 1<br>2<br>3<br>4<br>5 |
| 134 | Thinking about last week:<br>Have you done things with other girls? | Never..........................<br>Almost never..............<br>Sometimes.................<br>Almost always............<br>Always....................... | 1<br>2<br>3<br>4<br>5 |
| 135 | Thinking about last week:<br>Have you had fun with your friends? | Never..........................<br>Almost never..............<br>Sometimes.................<br>Almost always............<br>Always....................... | 1<br>2<br>3<br>4<br>5 |
| 136 | Thinking about last week:<br>Have you and your friends helped each other? | Never..........................<br>Almost never..............<br>Sometimes.................<br>Almost always............<br>Always....................... | 1<br>2<br>3<br>4<br>5 |
| 137 | Thinking about last week:<br>Have you been able to talk about everything with your friends? | Never..........................<br>Almost never..............<br>Sometimes.................<br>Almost always............<br>Always....................... | 1<br>2<br>3<br>4<br>5 |
| 138 | Thinking about last week:<br>Have you been able to rely on your friends? | Never..........................<br>Almost never..............<br>Sometimes.................<br>Almost always............<br>Always....................... | 1<br>2<br>3<br>4<br>5 |
| **Section 9: School and Learning** | | | |
| 139 | Thinking about last week:<br>Have you been happy at school? | Not at all.....................<br>Slightly.........................<br>Moderately..................<br>Very.............................<br>Extremely................... | 1<br>2<br>3<br>4<br>5 |
| 140 | Thinking about last week: | Not at all..................... | 1 |

| | | |
|---|---|---|
| | Have you got on well at school? | Slightly......................... 2<br>Moderately.................. 3<br>Very............................. 4<br>Extremely................... 5 |
| 141 | Thinking about last week:<br>Have you been satisfied with your teachers? | Not at all..................... 1<br>Slightly......................... 2<br>Moderately.................. 3<br>Very............................. 4<br>Extremely................... 5 |
| 142 | Thinking about last week:<br>Have you been able to pay attention? | Never.......................... 1<br>Almost never.............. 2<br>Sometimes................. 3<br>Almost always............ 4<br>Always....................... 5 |
| 143 | Thinking about last week:<br>Have you enjoyed going to school? | Never.......................... 1<br>Almost never.............. 2<br>Sometimes................. 3<br>Almost always............ 4<br>Always....................... 5 |
| 144 | Thinking about last week:<br>Have you got along well with your teachers? | Never.......................... 1<br>Almost never.............. 2<br>Sometimes................. 3<br>Almost always............ 4<br>Always....................... 5 |
| **Section 10: Bullying** | | |
| 145 | Thinking about last week:<br>Have you been afraid of other girls and boys? | Never.......................... 1<br>Almost never.............. 2<br>Sometimes................. 3<br>Almost always............ 4<br>Always....................... 5 |
| 146 | Thinking about last week:<br>Have other girls and boys made fun of you? | Never.......................... 1<br>Almost never.............. 2<br>Sometimes................. 3<br>Almost always............ 4<br>Always....................... 5 |
| 147 | Thinking about last week:<br>Have other girls and boys bullied you? | Never.......................... 1<br>Almost never.............. 2<br>Sometimes................. 3<br>Almost always............ 4<br>Always....................... 5 |
